# Physical Activity Enjoyment and Self-Efficacy As Predictors of Cancer Patients' Physical Activity Level

**DOI:** 10.3389/fpsyg.2016.00898

**Published:** 2016-06-21

**Authors:** Nadine Ungar, Joachim Wiskemann, Monika Sieverding

**Affiliations:** ^1^Institute of Psychology, Heidelberg UniversityHeidelberg, Germany; ^2^Division of Medical Oncology, National Center for Tumor Diseases Heidelberg and University Clinic HeidelbergHeidelberg, Germany

**Keywords:** physical activity enjoyment, self-efficacy, physical activity, cancer, affective factors, cognitive factors, intervention

## Abstract

**Background:** Physical activity (PA) can support cancer patients during medical treatment by reducing side-effects and increasing quality of life. However, PA levels mostly decline after diagnosis. Which factors can explain if patients are able to remain or even increase their PA level? Self-efficacy is an important cognitive factor that has been linked to cancer patients' PA across many studies. In contrast, affective factors such as PA enjoyment have rarely been examined. We compare the influence of self-efficacy and PA enjoyment on cancer patients' PA levels after completion of an exercise or stress-management intervention.

**Methods:** Outpatient cancer patients [*N* = 72; 54% female; *M* = 56 years, *SD* = 12.34; most with breast or colon cancer (34%, 15%)] were enrolled in the MOTIVACTION study, a 4-week intervention (1 h counseling followed by weekly phone calls), with pre-test (T1), post-test (T2), and a 10-week follow-up (T3). Participants were randomized to either an exercise intervention (emphasizing self-regulatory strategies for behavior change) or to a stress management intervention (coping and relaxation techniques). Sixty-seven patients remained in the study and completed the SQUASH assessment of PA, a measure of maintenance self-efficacy (7 items, Cronbach's α = 0.88) and PA enjoyment (2 items, Cronbach's α = 0.89). Regression analyses were calculated with PA level (at T2 and T3) as dependent variable and relative weight analyses were conducted. The study was registered at clinicalTrials.gov (unique identifier:NCT01576107; URL: https://clinicaltrials.gov/ct2/show/NCT01576107?term=motivaction&rank=1).

**Results:** Baseline self-efficacy and change in PA enjoyment significantly predicted cancer patients' PA level at T2 adjusting for baseline PA and type of intervention. Relative weight (RW) analysis revealed that PA enjoyment (baseline and change together) explained 34.3% of the dependent variable, self-efficacy (baseline and change) explained 38.4%. At follow-up, self-efficacy was still a significant predictor of PA (RW = 74.6%), whereas PA enjoyment was no longer a relevant factor (RW = 5.2%).

**Conclusion:** The affective factor PA enjoyment was equally important as self-efficacy for predicting cancer patient' PA level directly after completion of the intervention. Reasons for the reduced relevance at follow-up and a broader range of affective factors should be analyzed in future studies on cancer patients' PA level.

## Introduction

Regular physical activity (PA) has various beneficial effects for cancer patients both during and after medical treatment (Speck et al., 2010; Fong et al., 2012; Jones and Alfano, 2012). Current reviews and meta-analyses demonstrate, for example, positive effects on health-related quality of life (Mishra et al., 2012), cancer-related fatigue (Cramp and Byron-Daniel, 2012), depression (Craft et al., 2012), aerobic fitness (Jones et al., 2011), and muscle strength (Stene et al., 2013; Strasser et al., 2013). Cancer patients are recommended to engage in 150 min per week of at least moderate PA (Schmitz et al., 2010). However, it has been shown that only a minority of cancer patients meet these guidelines (Blanchard et al., 2008).

Various social cognitive theories—such as the Theory of Planned Behavior (TPB; Ajzen, 1991), the Social Cognitive Theory (SCT; Bandura, 1977, 1986), the Transtheoretical Model (Prochaska and DiClemente, 1983) or the Health Action Process Approach (Schwarzer, 2008)—try to explain why people do (not) engage in a health behavior such as PA. The TPB and the SCT have most frequently been applied to explain the low PA level of cancer patients (see Pinto and Ciccolo, 2011 for an overview). Self-efficacy is a core component of many social cognitive theories. As cancer patients face not only general barriers regarding PA such as bad weather, but also treatment related barriers such as fatigue (Brawley et al., 2002; Midtgaard et al., 2009; Blaney et al., 2013), self-efficacy is especially necessary in the oncological context to overcome these specific barriers. Self-efficacy turned out to be the best predictor of behavioral intention in most TPB studies in the field of PA and cancer (e.g., Karvinen et al., 2007; Keats et al., 2007; Speed-Andrews et al., 2012; Trinh et al., 2012) and often has an independent effect on PA behavior besides intention (e.g., Karvinen et al., 2007; Keats et al., 2007; Trinh et al., 2012). For example, there were moderate associations (*r* = 0.69/*r* = 0.43) between perceived behavioral control (similar to self-efficacy) and PA level/ intention in a cross-sectional study of 600 colorectal cancer survivors (Speed-Andrews et al., 2012). Similarly, in studies applying the SCT, self-efficacy is consistently identified as a psychosocial determinant of PA among cancer patients (e.g., Phillips and McAuley, 2013; Rogers et al., 2004). A meta-analysis by Stacey et al. (2015) summarizes behavior change studies using the SCT. It included twelve studies applying PA interventions based on the SCT to oncological patients. The meta-analysis found a significant intervention effect for increased PA levels (standardized mean difference = 0.33). Improvements in self-efficacy were in some studies associated with a following increase in PA (Pinto et al., 2005; Demark-Wahnefried et al., 2007; von Gruenigen et al., 2008; Ligibel et al., 2012).

Common theories of health behavior such as the TPB focus on cognitive constructs and do not explicitly incorporate affective factors, which can lead to a limited predictive power of these theories (McEachan et al., 2011; Conner et al., 2015). Current research tries to reduce this gap by including affective factors (Williams and Evans, 2014). PA enjoyment is one prominent and frequently applied affective component (see reviews by Rhodes et al., 2010; Nasuti and Rhodes, 2013) capturing experience and expectation of pleasure toward PA (Williams and Evans, 2014; Lewis et al., 2016).

In the context of cancer research, affective factors are very important, as many patients often experience psychological and emotional distress as well as depressive symptoms (Knobf, 2007; Jayadevappa et al., 2012; Jones et al., 2015). To deal with the disease many patients use various self-management strategies, whereby PA is the most commonly used one (Shneerson et al., 2015). Thereby, feeling self-determined regarding PA can increase positive affect (Brunet et al., 2013) and being passionate about activities positively affects emotional well-being (Burke et al., 2012).

Up to now, only a few studies have included PA enjoyment to predict cancer patients' PA level (e.g., Rogers et al., 2008, 2011; Charlier et al., 2013). PA enjoyment was mostly assessed with a one item measure (e.g., 2015; Rogers et al., 2011). Within these mostly correlational studies, PA enjoyment was always one of the strongest determinants of PA (Rogers et al., 2008; Charlier et al., 2013). For example, in a correlational study among head and neck cancer patients, task self-efficacy (*r* = 0.33), perceived barriers (*r* = −0.27), and PA enjoyment (*r* = 0.41) were the strongest correlates of PA (Rogers et al., 2008). In another cross-sectional study among 464 breast cancer survivors, PA enjoyment significantly explained leisure time PA (β = 0.2), but did not influence other domains of PA such as household or transportation (Charlier et al., 2013).

As self-efficacy as well as PA enjoyment have shown to be associated with PA, an important issue is the relative importance of affective factors compared to cognitive factors in explaining and predicting PA. This was the focus of a recent longitudinal study with healthy adults (mainly women), which investigated the relative importance and interrelationships between self-efficacy and PA enjoyment in predicting the initiation and maintenance of PA (Lewis et al., 2016). It turned out that PA enjoyment was a more powerful predictor for PA at a 12-month follow-up than self-efficacy. To explain this result, the authors tested several mediation models and found that PA enjoyment exerted its effects on PA levels through self-efficacy. Among people with a chronic disease such as cancer, the relative importance of self-efficacy and PA enjoyment has—to our knowledge—not been investigated so far.

If self-efficacy and PA enjoyment can lead to an increased PA level, improvements in these variables are likely to result in continued PA adherence (Morielli et al., 2016). Thus, it would be favorable if these two factors could be increased through an intervention as well. With respect to self-efficacy, Bandura's theory postulates four sources: mastery experience, vicarious experience, verbal persuasion and emotional arousal (Bandura, 1977, 2000). In a successful PA promotion intervention, a participant should experience mastery through feeling able to perform more PA now. The other three sources could be addressed in an intervention as well (e.g., vicarious experience through contact with a role model). In contrast to self-efficacy which is a “classic” and often studied cognitive construct, the affective construct PA enjoyment was introduced more recently and—at least to our knowledge—no theoretical assumptions have been formulated yet which factors should lead to an increase. However, first two pilot studies found improvements in PA enjoyment by a PA intervention (Rogers et al., 2011; Morielli et al., 2016). This leads to the question if PA enjoyment can be increased through a PA promotion intervention and which psychological, medical and sociodemograhpic variables are related to a change in PA enjoyment.

In the current study, we want to compare the influence of self-efficacy and PA enjoyment on cancer patients' PA level after completion of an exercise or stress-management intervention. The main objective is to investigate if the affective variable PA enjoyment predicts the PA level of cancer patients (at T2 and T3) over and above the cognitive variable self-efficacy (research question 1). Furthermore, we were interested to explore whether self-efficacy and PA enjoyment increased during the interventions (research question 2). Finally, additional analysis examine exploratively which factors are associated with changes in PA enjoyment.

## Methods

### Design

The MOTIVACTION (MOTivational InterVention enhancing physical ACTivity In ONcology patients) study consisted of two interventions (exercise and stress management) and assessments at baseline (T1), 4 weeks after the intervention (T2), and 10 weeks after T2 (T3). Patients were randomized to one of the two groups by being stratified by sex, age (i.e., < or ≥ 60 years), metastases (i.e., yes/no), and current chemotherapy (i.e., yes/no). The study protocol was approved by the ethic committee of the medical faculty in Heidelberg, Germany. All subjects gave written informed consent in accordance with the Declaration of Helsinki. The study was registered at clinicalTrials.gov (unique identifier: NCT01576107; URL: https://clinicaltrials.gov/ct2/show/NCT01576107?term=motivaction&rank=1).

### Participants

Patients of any cancer entity meeting the following inclusion criteria were eligible to participate in the study: receiving out-patient therapy (acute or maintenance therapy) or finished this therapy not longer than 6 months ago, being ≥ 18 years, being below the current guidelines to be moderate active at least for 150 min per week, and being able to follow the study instructions. Exclusion criteria were: planned rehabilitation or inpatient treatment during the next 8 weeks, wound healing not completed, bone metastases, and serious comorbidities or comorbidity-related limitations.

### Procedure

Participants were recruited at the National Center for Tumor Diseases in Heidelberg (Germany). In an initial (telephonic) meeting participants were screened if inclusion or exclusion criteria apply. Among others, patients were asked to rate their PA behavior in minutes during the last week. If the patients were less active than 150/min per week they were categorized as insufficiently active and could be enrolled in the study (if no other exclusion criteria occurred). After signing written informed consent participants were randomized either into the exercise or stress management group and received the baseline questionnaires. Both the exercise as well as the stress management intervention started immediately after returning baseline materials and consisted of an individual 1-h-counseling session, followed by three weekly telephone calls.

### Interventions

The exercise intervention included counseling based on the Health Action Process Approach (Schwarzer, 2008). Patients received a booklet with behavior-change techniques (e.g., action planning, coping planning) and various exercises for home-based training, exercise materials (e.g., stretch band for resistance training) as well as a diary to record exercise sessions. The stress management intervention consisted of relaxation techniques such as abdominal breathing, progressive muscle relaxation and cognitive coping techniques (Jacobsen et al., 2013). Patients received a booklet with stress management techniques, a CD with relaxation techniques as well as a diary to record stress management sessions. The procedure and content of the two interventions are described in detail in Ungar et al. (2016).

### Measures

*PA enjoyment* was assessed with two items based on Rogers et al. (2011, 2015). The two items were “I enjoy being regularly physically active” and “It is fun to engage in sport activities and regular PA”. A response scale from 1 (“not at all”) to 4 (“totally agree”) was used and a mean was calculated from the two items. Cronbach's alpha was good (α = 0.89).

*Maintenance self-efficacy* was assessed according to guidelines of the Health Action Process Approach (Schwarzer et al., 2003; Schwarzer, 2008). Seven items were used measuring the confidence with sticking to regular PA. An example item was “I am confident that I can permanently be regularly physically active even if I have side-effects (e.g., nausea) of the cancer-therapy” with a response format from 1 “not at all” to 4 “totally agree”. The internal consisty was good (Cronbach's α = 0.88) and a mean was used to aggregate the seven items.

*Physical activity* was measured using the slightly modified Short QUestionnaire to ASsess Health-enhancing physical activity (SQUASH) having good psychometric properties (Wendel-Vos et al., 2003; Wagenmakers et al., 2008).

The slightly modified questionnaire contained questions about physical activities related to commuting, household, leisure-time, and work. Every activity referred to the last 7 days and included three questions: days per week, average time per day and intensity (light, moderate, or vigorous). As the intervention focused on exercise behavior and not PA in general, we generated the variable “intended physical activity level” (iPAL). We classified the activities according to Caspersen et al. (1985) as exercise is “physical activity that is planned, structured, repetitive, and purposive in the sense that improvement or maintenance of one or more components of physical fitness is an objective” (p.128). iPAL therefore included all items of the domain leisure-time activities except gardening and odd work if participants reported an at least moderate intensity. These six items were: brisk (Nordic-) walking, bicycling, gymnastic or resistance training and three open-ended items called “sport activities.” The open-ended responses were excluded from analysis if the indicated activity was below a metabolic equivalent of 4 according to the Ainsworth compendium (Ainsworth et al., 2011).

### Data analysis

Change scores of self-efficacy and PA enjoyment where calculated by subtracting the respective T2 scores from the T1 scores (T2 minus T1). Descriptive statistics and bivariate Pearson correlations were conducted. Drop-out analyses were calculated to compare completers versus non-completers of the intervention using *t*-tests for metric and Chi-squared tests for nominal variables. The first research question was analyzed using linear regression models and relative weight analysis. Relative weight analysis estimates the relative importance of correlated determinants in a regression equation by using a new set of uncorrelated determinants that are maximally related to the original set of correlated determinants (Tonidandel et al., 2009). A relative weight is the amount of variance explained by a single determinant; all relative weights add up to 100%. For the second research questions, analyses of variances (ANOVAs) for repeated measures [between subject factor: type of intervention (exercise/stress); within subject factor: time (T1/T2/T3)] were computed with self-efficacy and PA enjoyment as dependent variables. Additional analyses were calculated using regression analysis explaining the dependent variable Δ_T1-*T*2_PA enjoyment.

Listwise deletion was used if necessary in all analyses, as there were only less than 2% of data missing. Analyses were carried out with IBM SPSS Statistics 22 IBM corp. NY, USA, and employed a significance level of *p* < 0.05.

## Results

### Sample and descriptives

The sample consisted of 72 cancer patients who were insufficiently active, that is not meeting guidelines of at least 150 min/week moderate PA at T1 (*M* = 47.22 min). They were randomized to the exercise intervention (*n* = 36) or to the stress management group (*n* = 36), with 67 completing the study (3 died, 2 drop-outs; details about the study flow can be found in Ungar et al., 2016). Participants were 52% female with a mean age of *M* = 55.45 years (*SD* = 12.62, range: 26–83 years). The majority had breast (33%), colorectal (12%), or prostate cancer (8%) with 33% having metastases and 37% having currently chemo-therapy (see Table 1).

**Table 1:** **Baseline characteristics of the sample (*N* = 67)**.

	***M***	**(*SD*)**	***N***	**(%)**
Age in years	55.45	12.62		
Female			35	52.2
Family-status				
Relationship			55	82.1
Single			6	9.0
Separated			6	9.0
Education level^a^				
Low			13	19.4
Middle			17	25.4
High			37	55.2
Occupation				
Full-time			13	19.4
Part-time			8	11.9
No occupation			46	68.7
Cancer entity				
Breast			22	32.8
Colorectal			8	11.9
Prostate			5	7.5
Others			27	43.5
Existance of metastases			22	32.8
Chemotherapy				
Currently			25	37.3
Completed			16	23.9
Radiotherapy				
Currently			10	14.9
Completed			14	20.9
Treatment at NCT			42	62.7

a *low: ≤ 9 years; middle: 10 years; high: 12 years or more*.

#### Baseline values in iPAL, self-efficacy and PA enjoyment

Descriptive results and bivariate correlations are shown in Tables 2, 3. The correlation between PA enjoyment and self-efficacy was moderate (*r* = 0.372, *p* = 0.003) at baseline. Mean values of iPAL (*M*_exercise_ = 52.14, *SD* = 101.95; *M*_stress-management_ = 41.84, *SD* = 92.47), PA enjoyment (*M*_exercise_ = 2.89, *SD* = 0.74; *M*_stress-management_ = 2.84, *SD* = 0.81), and self-efficacy (*M*_exercise_ = 2.61, *SD* = 0.50; *M*_stress-management_ = 2.59, *SD* = 0.59) did not differ significantly between the exercise intervention and the stress-management group before the start of the intervention (T1) (*p* > 0.05).

**Table 2:** **Descriptive statistics of study variables for the two intervention groups and for the whole sample**.

		**Exercise**		**Stress-management**		**Total**	
		***M***	**(*SD*)**	***M***	**(*SD*)**	***M***	**(*SD*)**
Intended physical	iPAL T1	52.14	101.95	41.84	92.47	47.22	96.94
Activity level (iPAL)^a^	iPAL T2	150.43	161.13	69.06	121.10	111.57	148.12
	iPAL T3	189.43	243.14	120.00	193.38	156.27	221.90
PA enjoyment^b^	PA enjoyment T1	2.89	0.74	2.84	0.81	2.87	0.77
	PA enjoyment T2	3.23	0.73	3.22	0.66	3.22	0.69
	PA enjoyment T3	3.15	0.87	3.22	0.73	3.18	0.80
Self-efficacy^b^	Self-efficacy T1	2.61	0.50	2.59	0.59	2.60	0.54
	Self-efficacy T2	2.75	0.56	2.74	0.61	2.74	0.58
	Self-efficacy T3	2.73	0.60	2.79	0.58	2.76	0.59
Change-scores	ΔiPAL T1-T2	98.29	201.14	27.22	118.60	64.35	169.49
	ΔiPAL T1-T3	137.29	249.60	78.16	194.63	109.05	225.33
	ΔiPAL T2-T3	39.00	228.11	50.94	203.97	44.70	215.36
	ΔPA enjoyment T1-T2	0.34	0.79	0.38	0.43	0.35	0.64
	ΔPA enjoyment T1-T3	0.26	0.97	0.36	0.82	0.31	0.90
	ΔPA enjoyment T2-T3	−0.08	0.74	0.00	0.74	0.04	0.73
	Δself-ef icacy T1-T2	0.14	0.56	0.15	0.62	0.14	0.58
	Δself-ef icacy T1-T3	0.12	0.66	0.20	0.40	0.16	0.55
	Δself-ef icacy T2-T3	−0.02	0.57	0.05	0.58	0.02	0.57

a In minutes per week (practiced during the last week);

b *On a scale from 1 to 4*.

**Table 3:** **Intercorrelations between study variables**.

		***n***	**2**	**3**	**4**	**5**	**6**	**7**	**8**	**9**	**10**	**11**	**12**	**13**	**14**	**15**	**16**	**17**	**18**	**19**	**20**	**21**	**22**	**23**
Intended	1. iPAL T1	67	0.09	0.18	0.25^◦^	0.12	0.14	0.16	0.05	0.08	−0.49^***^	−0.25^*^	0.13	−0.13	−0.04	0.07	−0.09	−0.08	0.03	0.05	−0.14	0.16	−0.03	−0.22^◦^
physical activity	2. iPAL T2	67		0.38^**^	0.2	0.35^**^	0.32^**^	0.23^◦^	0.42^***^	0.42^**^	0.82^***^	0.33^**^	−0.30^*^	0.17	0.15	0.03	0.16	0.24^◦^	0.00	0.28^*^	0.01	0.05	0.04	−0.18
level (iPAL)^a^	3. iPAL T3	67			0.09	0.15	0.19	0.42^**^	0.34^**^	0.36^**^	0.23^◦^	0.91^***^	0.77^***^	0.06	0.11	0.04	−0.09	0.00	0.02	0.16	−0.03	0.02	−0.05	−0.10
PA enjoyment^b^	4. PA enjoyment T1	62				0.62^***^	0.36^**^	0.37^**^	0.19	0.36^**^	0.05	0.00	−0.03	−0.51^***^	−0.53^***^	−0.23^◦^	−0.17	0.01	0.20	0.03	−0.16	−0.02	0.02	−0.19
	5. PA enjoyment T2	65					0.51^***^	0.27^*^	0.30^*^	0.31^*^	0.27^*^	0.11	−0.09	0.35^***^	−0.07	−0.40 ^**^	0.05	0.06	0.00	0.01	−0.24^◦^	−0.23^◦^	0.12	−0.03
	6. PA enjoyment T3	66						0.43^***^	0.27^*^	0.53^***^	0.20	0.13	−0.02	0.17	0.60^***^	0.59^***^	−0.10	0.14	0.26^*^	−0.05	−0.03	−0.18	−0.05	−0.09
Self-efficacy^b^	7. Self-efficacy T1	66							0.45^***^	0.54^***^	0.11	0.34^**^	0.26^*^	−0.15	0.07	0.15	−0.48^***^	−0.41^**^	0.10	−0.02	−0.08	−0.05	−0.05	−0.13
	8. Self-efficacy T2	67								0.52^***^	0.34^**^	0.31^**^	0.06	0.15	0.10	−0.05	0.57^***^	0.14	−0.49^***^	0.01	0.02	0.02	0.03	−0.02
	9. Self-efficacy T3	66									0.32^**^	0.32^**^	0.08	−0.07	0.16	0.23^◦^	0.04	0.55^***^	0.49^***^	−0.05	−0.05	−0.06	−0.10	−0.28^*^
Change-scores	10. ΔiPAL T1-T2	67										0.43^***^	−0.33^**^	0.24^◦^	0.17	0.00	0.19	0.26^*^	−0.02	0.21^◦^	0.09	−0.05	0.05	−0.04
	11. ΔiPAL T1-T3	67											0.71^***^	0.11	0.12	0.02	−0.05	0.04	0.00	0.13	0.03	−0.05	−0.04	−0.01
	12. ΔiPAL T2-T3	67												−0.05	0.01	0.02	−0.19	−0.16	0.02	−0.03	−0.04	−0.02	−0.08	0.02
	13. ΔPA enjoyment T1-T2	61													0.59^***^	−0.15	0.28^*^	0.07	−0.22^◦^	−0.04	−0.03	−0.22^◦^	0.16	0.26^*^
	14. ΔPA enjoyment T1-T3	61														0.71^***^	0.03	0.10	0.07	−0.04	0.12	−0.15	−0.05	0.09
	15. ΔPA enjoyment T2-T3	61															−0.15	0.09	0.28^*^	0.00	0.13	0.05	−0.18	−0.09
	16. Δself-efficacy T1-T2	66																0.52^**^	−0.55^***^	−0.04	0.07	0.06	0.10	0.12
	17. Δself-efficacy T1-T3	66																	0.44^***^	−0.07	0.04	−0.01	−0.06	−0.19
	18. Δself-efficacy T2-T3	66																		−0.06	−0.07	−0.08	−0.13	−0.27^*^
Other	19. Type of intervention	67																			0.04	0.10	0.10	−0.07
	20. Sex	67																				0.07	−0.10	0.18
	21. Age	67																					0.26^*^	0.02
	22. Metastases	67																						0.32^**^
	23. Chemo-therapy	67																					

a In minutes per week (practiced during the last week);

b On a scale from 1 to 4; type of intervention: 0 = stress-management, 1 = exercise; sex: 0 = male, 1 = female; metastases: 0 = no, 1 = yes; chemo-therapy: 0 = currently not, 1 = currently chemo-therapy;

◦ p < 0.10,

* *p < 0.05*.

** *p < 0.01*.

*** *p < 0.001*.

### Research question 1: Prediction of iPAL by PA enjoyment and self-efficacy

As was described in Ungar et al. (2016), iPAL increased from T1 to T2 and T3 in both intervention groups (exercise group: *M*_T1_ = 52.14, *SD*_T1_ = 101.95; *M*_T2_ = 150.43, *SD*_T2_ = 161.13; *M*_T3_ = 189.43, *SD*_T3_ = 243.14; stress-management group: *M*_T1_ = 41.84, *SD*_T1_ = 92.47; *M*_T2_ = 69.06, *SD*_T2_ = 121.10; *M*_T3_ = 120.00, *SD*_T3_ = 193.38). The first hierarchical regression analysis predicting iPAL at T2 could explain 22.8% of variance in the first step (including type of intervention, iPAL at T1, self-efficacy at T1 and Δ_T1-*T*2_ self-efficacy as independent variables) and further 5% in the second step (including additionally PA enjoyment at T1 and Δ_T1-*T*2_PA enjoyment). Self-efficacy (at T1 and Δ_T1-*T*2_) was a positive significant predictor, and Δ_T1-*T*2_PA enjoyment could predict iPAL at T2 over and above this cognitive influence (β = 0.32). Relative weight analysis revealed that self-efficacy and PA enjoyment were of about equal importance in predicting iPAL at T2 (see Table 4 for detailed statistics).

**Table 4:** **Hierarchical regression analysis predicting intended physical activity level (iPAL) at T2 and T3 (*N* = 61)**.

**Step**	**Predictor**	**β_step 1_**	**β_step 2_**	**RW_%_**
**AV = iPAL at T2**				
1:	Type of intervention^a^	0.32^**^	0.33^**^	25.8
	iPAL T1	0.22^◦^	0.20^◦^	1.5
	Self-efficacy T1^b^	0.35^**^	0.27^*^	22.1
	Δself-efficacy T1-T2	0.29^*^	0.20	16.3
2:	PA enjoyment T1^b^		0.24^◦^	16.8
	ΔPA enjoyment T1-T2		0.32^*^	17.5
*R*^2^ for model		0.28	0.36	
Adjusted *R*^2^		0.23	0.28	
Δ*R*^2^ for last step		0.28^**^	0.08^◦^	
**AV = iPAL at T3**				
1:	Type of intervention^a^	0.11	0.11	9.9
	iPAL T1	0.12	0.14	10.3
	Self-efficacy T1^b^	0.46^**^	0.47^**^	69.1
	Δself-efficacy T1-T2	0.12	0.09	5.5
2:	PA enjoyment T1^b^		−0.05	2.9
	ΔPA enjoyment T1-T2		0.11	2.3
*R*^2^ for model		0.22	0.24	
Adjusted *R*^2^		0.16	0.15	
Δ*R*^2^ for last step		0.22^**^	0.02	

a Type of intervention: 0 = stress-management, 1 = exercise;

b On a scale from 1 to 4;

° p < 0.10

* *p < 0.05*.

** *p < 0.01*.

The second hierarchical regression analysis predicting iPAL at T3 revealed a differing finding. In the first step (including type of intervention, iPAL at T1, self-efficacy at T1 and Δ_T1-*T*2_ self-efficacy as independent variables) 16.4% of variance could be explained and only self-efficacy at T1 had a significant effect (β = 0.457, *t* = 3.305, *p* = 0.002). In the second step, PA enjoyment (at T1 as well as Δ_T1-*T*2_) did not add any variance and was no significant predictor (see Table 4).

### Research question 2: Changes in self-efficacy and PA enjoyment

Two ANOVAS with repeated measures showed that both PA enjoyment [*F*_(2, 116)_ = 7.317, *p* = 0.001] and also marginally self-efficacy [*F*_(2, 126)_ = 2.690, *p* = 0.072] increased across the three measurement points (see Table 2). Looking at the effect sizes, the increase of PA enjoyment was almost three times larger than the increase in self-efficacy ($\mu_{\text{PA enjoyment}}^{2}=0.112$ versus $\mu_{\text{self-efficacy}}^{2}=$ 0.041). The interaction time by type of intervention (exercise versus stress management) and the main factor type of intervention had no effect in both analyses [PA enjoyment: *F*_condition(1, 58)_ = 0.029, *p* = 0.866, *F*_interaction(2, 116)_ = 0.040, *p* = 0.961; self-efficacy: *F*_condition(1, 63)_ = 0.012, *p* = 0.915, *F*_interaction(2, 126)_ = 0.150, *p* = 0.860]. Thus, the factor type of intervention was left out in an additional analysis yielding very similar results [PA enjoyment: *F*_(2, 118)_ = 7.412, *p* = 0.001, μ^2^ = 0.112; self-efficacy: *F*_(2, 128)_ = 2.708, *p* = 0.71, μ^2^ = 0.041]. *Post-hoc* analyses revealed that the significant increase in PA enjoyment and marginally in self-efficacy only took place between T1 und T2 (*p*_PA enjoyment_ < 0.001; *p*_self-efficacy_ = 0.091), thus during the intervention. The difference to baseline remained significant at T3 (*p*_PA enjoyment_ = 0.006; *p*_self-efficacy_ = 0.025).

### Additional analyses: Factors related to change in PA enjoyment

To examine factors being associated with the significant increase in PA enjoyment (between T1 and T2) at first bivariate correlations were regarded (see Table 3). All variables from T1 and T2 which correlated at least marginally (*p* < 0.10) with Δ_T1-*T*2_PA enjoyment [i.e., age, change (T1-T2) in iPAL, chemo-therapy status, PA enjoyment at T1, change (T1-T2) in self-efficacy] were included in a regression analysis as independent variables (dependent variable = Δ_T1-*T*2_PA enjoyment). The regression analysis could explain 37% of the variance in the change of PA enjoyment. Low PA enjoyment at T1 (β = −0.472, *t* = −4.437, *p* < 0.001), a high increase in iPAL between T1 and T2 (β = 0.249, *t* = 2.389, *p* = 0.020) and a younger age (β = −0.215, *t* = −2.096, *p* = 0.041) were significant predictors of an increase in PA enjoyment.

## Discussion

Increasing evidence hints to the important role of affective factors in the process of behavior change (see introduction). Positive affects might be especially important in an oncological setting, as a cancer diagnosis and oncological treatment is linked to emotional distress and negative emotions (Janz et al., 2013; Deimling et al., 2015). Research shows that being passionate about activities is important for enhancing well-being and lowers cancer worry (Burke et al., 2012). Our analysis focused on the role of PA enjoyment. With regard to #PA of cancer patients, PA enjoyment was compared to the classical cognitive factor self-efficacy.

The main result of our study was that baseline PA enjoyment as well as change in PA enjoyment (T1-T2) predicted the iPAL of cancer patients directly after the intervention. A relative weight analysis showed that the effect of PA enjoyment was comparable in its size to the one of the well-established cognitive factor self-efficacy. This is in line with previous studies showing the prominent role of PA enjoyment in predicting PA behavior in healthy adults (e.g., Rhodes et al., 2010; Nasuti and Rhodes, 2013; Lewis et al., 2016). As many people react with distress and depressive symptoms when being confronted with a cancer diagnosis (Jayadevappa et al., 2012; Jones et al., 2015), affective factors might play an especially important role in the oncological setting. Being physically active is one of several self-management strategies cancer patients use to actively deal with the disease (Shneerson et al., 2015). Research has found that regular PA can reduce depressive symptoms and improve the affective mental state (Craft et al., 2012; Mishra et al., 2012). A few mostly correlational studies have already shown that PA enjoyment is an important predictor of PA behavior also in oncological settings (Rogers et al., 2008; Charlier et al., 2013). In these studies PA is compared to other social cognitive factors in explaining cancer patients' PA. PA enjoyment always showed significant mostly moderate associations with PA (e.g., *r* = 0.4 in the study by Rogers et al., 2008).

While we found a significant effect of PA enjoyment on iPAL directly after intervention (at T2), surprisingly, PA enjoyment did not have any influence on iPAL at the 10-week follow up (T3). This is in contrast to findings of Lewis et al. (2016), who compared the relative importance of PA enjoyment and self-efficacy in a longitudinal study with a 12 months follow up in healthy adults, and found that self-efficacy was no longer a significant predictor of PA level when PA enjoyment was controlled. One reason for the more relevant role of PA enjoyment in this study by Lewi et al. compared to ours might be that Lewi's study assessed PA enjoyment more detailed with the 18-item PACES scale (Kendzierski and DeCarlo, 1991). However, our result is comparable to—to our knowledge—the only previous PA promotion trial among oncological patients in which the role of PA enjoyment was examined among 41 breast cancer survivors (Rogers et al., 2011). They found an increase in PA enjoyment directly post intervention as well, but PA enjoyment did not mediate the intervention effect on PA at a 3 months follow-up (barrier self-efficacy on the other hand showed this mediating effect).

If results of our study and the one by Rogers et al. (2011) can be replicated, reasons have to be examined why PA enjoyment is a significant predictor of long term PA in the healthy population (Lewis et al., 2016) but not in an oncological setting. For patients living with a serious disease such as cancer, cognitive factors might be more relevant for maintaining their PA level than affective factors in the long run. For example, in an unpublished survey of our working group among 193 cancer patients of various entities we found that only 13% of patient engaged in physical activities they preferred to do. Thus, cancer patients often cannot carry out the activities they really enjoy (for example playing soccer), but have to choose a suitable type of PA according to the limitations of their illness and the treatment(s) (for example Nordic Walking). Moreover, treatment related barriers (such as side-effects, fatigue, etc.) might make it additionally difficult for oncological patients to maintain their PA level compared to healthy adults (Brawley et al., 2002; Midtgaard et al., 2009; Blaney et al., 2013), pointing out the prominent role of barrier self-efficacy for long term PA.

Results of our study furthermore showed an increase in PA enjoyment and also marginally in self-efficacy during a 4-week behavior change intervention (in an exercise intervention as well as in a stress management group). The increase in self-efficacy is in line with Banduras theory (Bandura, 1977, 2000), postulating that feeling mastery experience regarding PA during an intervention should yield an increase in self-efficacy. Compared to the baseline values the increase in PA enjoyment was nearly three times as much as the one of self-efficacy ($\mu_{\text{PA enjoyment}}^{2}=0.11$; $\mu_{\text{self-efficacy}}^{2}=$ 0.04). This is very interesting, as there is no theory explaining changes in PA enjoyment. To our knowledge, only one very recent pilot study among 18 rectal cancer patients has examined increases in PA enjoyment while participating in an exercise program (Morielli et al., 2016). Because of the lack in theoretical background as well as empirical evidence, we exploratively analyzed factors being associated with the increase in PA enjoyment: Increases in PA enjoyment were highest in younger patients, in participants with low scores on PA enjoyment at baseline and in participants who were able to considerably increase their iPAL during the intervention. Looking at this last factor leads to the assumption that changes in PA enjoyment might be related to mastery experience as well. Other potential factors like social aspects of exercising together (including the sources of self-efficacy *vicarious experience* and *personal persuasion*) or physical arousal during exercising (similar to the last source of self-efficacy) might be relevant as well and should be examined in future research. Moreover, the characteristics being a younger patient and having low scores on PA enjoyment should be prescreened in future studies and in current counseling initiatives to define the most valuable group for PA behavior change (trials) in cancer patients. Furthermore, within these patient groups the value of affective exercise components like competition-orientated tasks should be integrated in future study designs.

Our study has several limitations. Firstly, PA enjoyment has been measured with two items only. Although it has a good internal consistency and is based on a measure used in other oncological studies (e.g., Rogers et al., 2011, 2015), a multi-item measure such as the PA enjoyment Scale (PACES; Kendzierski and DeCarlo, 1991) would be preferable and would allow more differentiated analysis. As PA enjoyment was compared to self-efficacy which was assessed much more in detail, our results might be affected by this unequal assessment methods. Secondly, analyses were based on self-report measure for iPAL only and were restricted on exercise-related physical activities instead of PA in general. Thirdly, the focus of this article was limited to self-efficacy and PA enjoyment. Other affective factors besides PA enjoyment (e.g. affective attitudes, affective outcome expectancies, fear of PA) have not been taken into account and might be more relevant for explaining long term PA behavior of cancer patients. Lastly, the intervention study did not include a usual care control group. Results showed an increase in PA enjoyment in both the exercise and the stress management group. Thus, it might be that PA enjoyment increased through the social contact with the intervention staff. However, people of the stress management group also engaged more frequently in PA after the intervention. Thus, probably not the intervention itself but the increase in PA predicted if participants enjoyed PA more after the intervention (Liao et al., 2015).

Future research should bring the single constructs—self-efficacy and PA enjoyment—into a broader context. For example, a classical social cognitive theory including self-efficacy (e.g., TPB or SCT) could be compared to a theory focusing on affective factors. One of the rare affective theories called “affect and health behavior framework” was proposed by Williams and Evans (2014). This complex model suggests pathways—for example automatic and reflective affect processing or affectively charged motivation—through which affect related concepts interrelate and influence health behavior. It would be interesting to test this framework in the PA and cancer domain empirically.

In conclusion, our study was the first study examining the relative importance of PA enjoyment in comparison to the classical cognitive factor self-efficacy in a PA promotion intervention for cancer patients. At least in the short term affective factors such as PA enjoyment seem to play an important role for cancer patients to increase their PA level.

## Author contributions

NU contributed substantially to the conception and design of the work, performed statistical analyses and drafted the manuscript. JW supervised and supported the conception, design and implementation of the study and revised the work critically. MS supervised and supported the conception and design of the study, interpreted the data of the work and revised the work critically. All authors read and approved the final manuscript.

### Conflict of interest statement

The authors declare that the research was conducted in the absence of any commercial or financial relationships that could be construed as a potential conflict of interest.
